# Influenza A Virus Infection Activates NLRP3 Inflammasome through Trans-Golgi Network Dispersion

**DOI:** 10.3390/v14010088

**Published:** 2022-01-05

**Authors:** Kannu Priya Pandey, Yan Zhou

**Affiliations:** 1 Vaccine and Infectious Disease Organization (VIDO), University of Saskatchewan, Saskatoon, SK S7N 5E3, Canada; kannu.pandey@usask.ca; 2 Vaccinology and Immunotherapeutics Program, School of Public Health, University of Saskatchewan, Saskatoon, SK S7N 2Z4, Canada

**Keywords:** influenza A virus, NLRP3 inflammasome, trans-Golgi network, M2 ion channel protein

## Abstract

The NLRP3 inflammasome consists of NLRP3, ASC, and pro-caspase-1 and is an important arm of the innate immune response against influenza A virus (IAV) infection. Upon infection, the inflammasome is activated, resulting in the production of IL-1β and IL-18, which recruits other immune cells to the site of infection. It has been suggested that in the presence of stress molecules such as nigericin, the trans-Golgi network (TGN) disperses into small puncta-like structures where NLRP3 is recruited and activated. Here, we investigated whether IAV infection could lead to TGN dispersion, whether dispersed TGN (dTGN) is responsible for NLRP3 inflammasome activation, and which viral protein is involved in this process. We showed that the IAV causes dTGN formation, which serves as one of the mechanisms of NLRP3 inflammasome activation in response to IAV infection. Furthermore, we generated a series of mutant IAVs that carry mutations in the M2 protein. We demonstrated the M2 proton channel activity, specifically His37 and Trp41 are pivotal for the dispersion of TGN, NLRP3 conformational change, and IL-1β induction. The results revealed a novel mechanism behind the activation and regulation of the NLRP3 inflammasome in IAV infection.

## 1. Introduction

Inflammation is initiated when the innate immune system recognizes invading pathogens or molecules from tissue injury through pattern recognition receptors (PRRs) including nucleotide-binding domain and leucine-rich repeat-containing proteins (NLRs) [1]. These PRRs recognize various pathogen-associated molecular patterns (PAMPs) and danger-associated molecular patterns (DAMPs) upon the induction of pathogens or other foreign materials [2]. Among these sensors, a member of the NLRs called the NLR family pyrin domain-containing protein 3 (NLRP3) can detect various PAMPs and DAMPs and had been extensively studied [3]. NLRP3, along with the adaptor molecule ASC [apoptosis-associated speck-like protein containing a caspase-activation and recruitment domain (CARD); also known as PYCARD] and the cysteine protease pro-caspase-1 form a three-part molecule known as the inflammasome [4]. It has been widely appreciated that the activation of the inflammasome occurs in two steps: priming and activation. Signal 1 or priming includes the recognition of PAMPs leading to the transcriptional upregulation of various components of the NLRP3 inflammasome, including NLRP3, pro-caspase-1, pro-IL-1β, and pro-IL-18. Signal 2 or the activation step, which can be activated by K+ efflux, Ca+ flux, lysosomal disruption, mitochondrial reactive oxygen species (mtROS) production resulting from pathogen infection or DAMP exposure, leads to NLRP3 self-oligomerization, conformational change, clustering of the PYD domain and subsequent recruitment of ASC [5]. This forms a speck-like structure upon visualizing in the microscope and may act as an indicator of the activation of the inflammasome [6]. ASC then interacts with pro-caspase-1 leading to the auto cleavage and formation of the active caspase-1. The activated caspase-1 then cleaves the pro-IL-1β and pro-IL-18, resulting in the secretion of mature proinflammatory cytokines [7].

Influenza A virus (IAV) can be sensed by PRRs such as the toll-like receptors (TLRs) and the retinoic acid-inducible gene I (RIG-I)-like receptors. Sensing of IAV by the PRRs leads to the expression of the various components of the inflammasome [8]. NLRP3 is known to recognize IAV infection and to mediate a host response that assists in clearing infection and aids in protection. However, dysregulation of NLRP3 will result in an excessive and prolonged immune response, which will enhance the disease burden [9]. Various IAV proteins are known to initiate the activation of the NLRP3 inflammasome. PB1-F2 protein is known to activate the NLRP3 inflammasome and to induce IL-1β secretion contributing to IAV pathogenesis [10]. The M2 protein is reported to facilitate inflammasome activation by localization to the acidified Golgi apparatus, promoting proton efflux [11]. Despite extensive explorations, the molecular mechanisms by which NLRP3 activation in response to IAV infection remain largely unknown.

Most recently, a novel mechanism of NLRP3 being activated by stress molecules is reported. In the presence of nigericin, the trans-Golgi network (TGN) disperses into small puncta-like structures where the NLRP3 is recruited and activated [12]. Here, we aimed to understand whether IAV infection leads to the activation of the NLRP3 inflammasome through TGN dispersion. We observed that IAV infection could not only induce the formation of dTGN but also lead to the co-localization of NLRP3 at the site of dTGN and the recruitment of ASC. Moreover, mutant IAVs carrying mutations at amino acids 37 and 41 in M2 protein reduced their ability to induce dTGN, NLRP3 conformational change, and IL-1β production. Our results revealed a novel mechanism behind the activation and regulation of the NLRP3 inflammasome in IAV infection. The knowledge gained in this study contributes to a better understanding of the molecular mechanism of NLRP3 inflammasome activation and regulation. A balanced regulation of NLRP3 inflammasome activation will aid the host to clear the IAV infection.

## 2. Materials and Methods

### 2.1. Cells and Viruses

Madin-Darby canine kidney (MDCK) cells were maintained in minimum essential medium (MEM) (M4655; Sigma-Aldrich, St. Louis, MO, USA); human embryonic kidney (HEK293T) and Henrietta Lacks (HeLa) cell lines were maintained in Dulbecco’s Modified Eagle’s Medium (DMEM) (D5796; Sigma-Aldrich, St. Louis, MO, USA). The cells were supplemented with 10% fetal bovine serum (FBS) (16000-044; Thermo Fisher, Mississauga, ON, Canada) and 50 μg/mL gentamicin in a 5% CO_2_ incubator at 37 °C. For culturing the NLRP3-GFP stable cell line, 0.5 μg/mL and 2 μg/mL of puromycin (ant-pr-1; Invitrogen, Carlsbad, CA, USA) was used as the selection antibiotic for HEK293T and HeLa cell lines, respectively.

Porcine alveolar macrophages (PAMs) were isolated from the bronchial lung lavage collected from 4–6-week-old piglets as previously described [13]. These cells were cultured in HyClone RPMI-1640 medium (SH30027.01; GE Healthcare, Chicago, IL, USA) supplemented with 20% FBS, 50 µg/mL gentamycin, and 1× antibiotic-antimycotic (15240-062; Thermo Fisher, Waltham, MA, USA).

Wild-type IAVs used in this study are swine influenza A/swine/Saskatchewan/18789/2002/H1N1 (Sk02) and human pandemic influenza A/Halifax/210/2009/H1N1 (Hf09) virus. They were grown in MDCK cells in the presence of 0.2% BSA (A7030; Sigma-Aldrich, St. Louis, MO, USA) and 1 μg/mL of L-[(toluene-4-sulfonamide)-2-phenyl]ethyl chloromethyl ketone (TPCK)- trypsin. Mutant M2 IAVs and NS reassortant M2 mutant viruses, namely Hf09 M2_H37C_, Hf09 M2W_41C_, Hf09 M2_H37C-W41C_, Hf09 NS_Sk02_ M2_H37C_, Hf09 NS_Sk02_ M2_W41C_, and Hf09 NS_Sk02_ M2_H37C-W41C_ were propagated in MDCK cells stably expressing M2 protein (MDCK-M2) as previously described [14]. The MDCK-M2 cells, expressing M2 protein derived from Hf09 WT were generated using a lentivirus system as previously described [15]. These MDCK-M2 cells were grown in MEM, supplemented with 10% FBS, 2 μg/mL of puromycin, and 50 μg/mL gentamicin in a 5% CO_2_ incubator at 37 °C. The Hf09 NS_Sk02_ WT virus was propagated in MDCK cells as previously described.

### 2.2. Plasmids

Flag-tagged porcine NLRP3 was cloned into pcDNA-GFP vector with a short linker (GPVAT) occurring between NLRP3 and GFP sequences [12]. For the preparation of a stable cell line expressing Flag-NLRP3-GFP in HEK293T and HeLa cells, plasmid pEB7.8 Flag-NLRP3-GFP was used. For the formation of NLRP3-ASC puncta, pcDNA3.1-ASC was used [16].

For the development of the mutant M2 viruses, pHW-Hf09-M underwent site-directed mutagenesis using specific primers. The resulting plasmid, pHW-Hf09 M2_H37C_, pHW-Hf09 M2_W41C_, pHW-Hf09 M2_H37C-W41C_, encode mutations on the M2 protein at amino acids 37 and 41, both located on the transmembrane domain. The sequence of the plasmid was confirmed by DNA sequencing to ensure only the desired mutations were introduced by PCR. The plasmids generated were used to produce three mutant viruses, Hf09 M2_H37C_, Hf09 M2_W41C_, and Hf09 M2_H37C-W41C_, through the use of the eight-plasmid reverse genetics system, as previously described [17].

To generate Hf09 NS_Sk02_ M2 mutant viruses namely, Hf09 NS_Sk02_ M2_H37C_, Hf09 NS_Sk02_ M2_W41C_, and Hf09 NS_Sk02_ M2_H37CW41C_, The NS segment of Hf09 was replaced by the NS segment of Sk02 in the eight-plasmid reverse genetic system, as described above.

### 2.3. Antibodies and Reagents

Antibodies namely, rabbit polyclonal NP and NS1 antibodies were produced in our laboratory [18]. The commercial antibodies used in this study are as follows: Goat anti-porcine IL-1β antibody (BAF681, R&D Systems, Minneapolis, MN, USA); rabbit anti-porcine caspase-1 (p20) antibody (PAB592Po01; Cloud-Clone Corp, Houston, TX, USA); mouse anti-β-actin antibody (3700; CST, Kansas City, MO, USA), mouse anti-FLAG M2 antibody (F3165; Sigma-Aldrich, St. Louis, MO, USA); mouse monoclonal anti-Myc tag antibody (2276; CST); mouse polyclonal anti-TGN38 (MA3-063; Invitrogen, Carlsbad, CA, USA); rabbit polyclonal anti-TGN46 (PA1-1069; Invitrogen); goat polyclonal anti-IAV (AB1074; EMD Millipore, Toronto, ON, Canada). Secondary antibodies used in the study were: IRDye 680RD donkey anti-rabbit (926-68073; LI-COR Biosciences, Lincoln, NE, USA), IRDye 800CW donkey anti-mouse (926-32212; LI-COR Biosciences), and IRDye 800CW donkey anti-goat (926-32214; LI-COR Biosciences); donkey anti-rabbit Alexa Fluor 647 (A-31573; Invitrogen); donkey anti-rabbit Alexa Fluor 546 (A10040; Invitrogen) and donkey anti-goat Alexa Fluor 633 (A-21082; Invitrogen). Please note, as the anti-TGN38 antibody was discontinued, we completed the study using the anti-TGN46 antibody where applicable.

The following reagents were used in our study: lipopolysaccharide (LPS) (L3024; Sigma-Aldrich), Nigericin (N7143, Sigma-Aldrich), Amantadine (A1260, Sigma-Aldrich); 4′,6-diamidino-2-phenylindole (DAPI) (D1306; Invitrogen), Coelenterazine-h (C6780, Invitrogen) and EDTA-free protease inhibitor cocktail tablets (4693132001; Roche, Calgary, AB, Canada). Transfection was conducted using TransIT-LT1 transfection reagent (MIR2300; Mirus Bio, Madison, WI, USA) as instructed by the manufacturer.

### 2.4. Virus Growth Kinetics and PAMs Infection

For viral growth kinetics, MDCK or MDCK-M2 cells were seeded at 2 × 10^5^ cells in a 6-well plate and were infected with WT or M2 mutant or NS_Sk02_ M2 mutant viruses at an MOI of 0.001. Cell-free supernatants were collected at various time points (12 h, 24 h, 36 h, 48 h, 60 h, and 72 h). These supernatants were then analyzed for their viral titers by plaque assay.

For PAMs infection, PAMs isolated from each piglet were tested for the background level of IL-1β by ELISA. Only batches with a limited level of IL-1β were chosen for viral infection. PAMs were seeded at 1 × 10^6^ cells per well in a 24-well plate and infected with the respective virus at MOI of 1 for 24 h. The cell-free supernatant was harvested for IL-1β ELISA [16] and the cells harvested were subjected to Western blotting.

### 2.5. NLRP3 Inflammasome Reconstitution and Viral Infection Assay

HEK293T cells were seeded at 2.5 × 10^5^ cells per well on a 24-well plate for 24 h and transfected with pcDNA-NLRP3 or pCMV-Flag-NLRP3 or pcDNA-Flag-NLRP3-GPVAT-GFP (30 ng), pcDNA-ASC (10 ng), pCMV-Flag-pro-caspase-1 (20 ng), and pcDNA-pro-IL-1β (100 ng). At 12 h post-transfection (h.p.t.), cells were infected with viruses at an MOI of 5. At 18 h post-infection (h.p.i), cell-free supernatant was harvested for porcine IL-1β ELISA.

For infection with IAV NS_Sk02_ reassortant viruses, HEK293T cells were seeded at 2.5 × 10^5^ cells per well on a 24 well plate for 24 h and transfected with pcDNA-NLRP3 (30 ng), pcDNA-ASC (10 ng), pCMV-Flag-pro-caspase-1 (20 ng), and pcDNA-pro-IL-1β (100 ng). At 12 h.p.t., cells were infected with viruses at an MOI of 5. At 12 h.p.i, cell-free supernatant was harvested for porcine IL-1β ELISA.

### 2.6. Porcine IL-1β ELISA

Immunol 2 HB-U plates (3655; Thermo Fisher, Roskilde, Denmark) were coated with 2 µg/mL of mouse monoclonal anti-porcine IL-1β antibody in phosphate-buffered saline (PBS) at room temperature overnight. All of the following steps were conducted at room temperature, and all reagents or samples were applied at 100 μL per well. The plates were washed four times with Tris-buffered saline (TBS) with 0.05% Tween 20 (TBST) between each step. Blocking was performed with 1% BSA in PBS for 1 hr. A two-fold serial dilution of recombinant porcine IL-1β protein (source) in diluent (0.1% BSA in TBST) was used as the standard. The plates were incubated with the standard or the samples for 2 h at room temperature, followed by incubation with 50 ng/mL of goat polyclonal anti-porcine IL-1β biotinylated antibody in diluent for 1 h. This was followed by the incubation with alkaline phosphatase streptavidin diluted to 5000 times in diluent for 1 h. For color development, the plates were incubated with 1mg/mL of p-nitrophenyl phosphate in diethanolamine buffer (PNPP) (1M diethanolamine, 0.5 M MgCl_2_, PH 9.8) until the optical density of the first dilution of the standard reached around 2.0. The plates were read at 405 nm with a reference at 490 nm using an xMark microplate absorbance spectrophotometer (Software: Microplate Manager 6 software) (Bio-Rad, Hercules, CA, USA).

### 2.7. Western Blotting

Cell lysates were subjected to SDS-PAGE followed by blotting on nitrocellulose membranes. The membranes were blocked with 5% skim milk in 1× TBST for 1 h and incubated with primary antibodies in 1× TBST at 4 °C overnight. The membranes were further incubated with secondary antibodies in 1× TBST at room temperature for 1 h and were scanned with an Odyssey Infrared Imager (LI-COR Biosciences).

### 2.8. Immunofluorescence Microscopy

HEK293T or HeLa cells or HEK293T stably expressing NLRP3-GFP or HeLa cells expressing NLRP3-GFP were seeded at 3 × 10^4^ cells per well on a Nunc^TM^ LabTek^TM^ II CC2^TM^ chamber slide (154941PK; Thermo Fisher). Where required, the cells were transfected with 500 ng of pcDNA-Flag-NLRP3-GFP and 200 ng pcDNA-ASC-Myc, pcDNA-Myc or pcDNA-GFP and incubated for 24 h before stimulating with LPS (200 ng/mL) or infected with the corresponding viruses at an MOI of 10. The cells were then incubated for the period specified. The slide was then washed with PBS and fixed with 4% paraformaldehyde for 10 min and permeabilized with 0.1% Saponin (47036; Sigma) in 5% BSA for 5 min at room temperature. The cells were probed with primary antibodies diluted at 1:1200 in 5% BSA + 0.1% Saponin overnight at 4 °C followed by incubation with 1:1200 ratio of secondary antibodies for 1 h at room temperature. The cells were washed with Dulbecco’s phosphate-buffered saline (DPBS) between each step. Cells were counterstained with DAPI for 5 min at room temperature where applicable and the slide was mounted with ProLong Diamond Antifade Mountant (P36961; Invitrogen) overnight. Images were visualized by a confocal laser scanning microscope (LI-COR).

### 2.9. NLRP3 Oligomerization

HEK293T cells were seeded at 5 × 10^5^ cells per well in a 6-well dish. The cells were transiently transfected with 1μg of pCMV-3×Flag-NLRP3 for 48 h and then mock-infected or infected with Hf09 WT at MOI of 5 for 12 h. After infection, the cells were washed once with ice-cold PBS and then lysed in ice-cold native lysis buffer (20 mM Bis-tris, 500 mM ε-aminocaproic acid, 20 mM NaCl, 10% (*w*/*v*) glycerol, 0.5% digitonin, 0.5 mM Na_3_VO_4_, 1 mM PMSF, 0.5 mM NaF, 1 × EDTA-free Roche protease inhibitor cocktail, pH 7.0) for 15 min on ice [19]. Cell lysates were clarified by centrifugation at 20,000× *g* for 30 min at 4 °C; Proteins were separated in 6% native PAGE and then analyzed by Western blot.

### 2.10. TGN Fractionation

Three plates (10 cm in diameter) of HEK293T cells were transfected with 1 µg pCMV-3×Flag–NLRP3 for 36 h and were then infected with Hf09 WT at an MOI of 5 for 4 h. The cells were then homogenized using a Dounce homogenizer in isotonic buffer [0.25 M sucrose, 10 mM Tris-HCl (pH 7.5), 10 mM KCl, 1.5 mM MgCl_2_, and protease inhibitor cocktail and centrifuged at 1000× *g* for 5 min to remove the nucleus pellet (P1)]. The supernatant (S1) was further centrifuged at 5000× *g* for 10 min to obtain a heavy membrane fraction (pellet, P5), while this supernatant (S5) was centrifuged at 100,000× *g* for 20 min to separate the light membrane fraction (pellet, P100) from the cytosol fraction (supernatant, S100). P5 and P100 were washed with isotonic buffer once and resuspended in the same buffer. The P5 fraction was then used for sucrose gradient ultracentrifugation separately [12].

### 2.11. BRET Assay

HEK293T cells were seeded at 1 × 10^3^ cells in a poly-L-Lysine coated 96-well flat bottom plate. The cells were transfected with either pCMV-rLuc or pcDNA3.1-YFP-NLRP3-Luc (kindly provided by Dr Y. He, Wayne State University School of Medicine, Detroit, MI, USA) for 24 h. The signal from BRET was read after adding 5 μM of coelenterazine-h for 30 min and then the cells are infected with virus at an MOI of 1. Luminescence was detected in 15 min intervals at 37 °C in a GloMax Explorer multiplate reader (Promega, Madison, WI, USA) using two filters for emission at 485 ± 20 nm and 530 ± 20 nm. The BRET ratio was calculated as the difference between the 528 nm and 485 nm emission ratio of R-Luc and YFP-NLRP3 fusion protein and the 530 nm and 485 nm emission ratio of the R-Luc protein alone. Results are expressed in milli-BRET (mBU) units normalized to a basal signal [19].

### 2.12. Statistical Analysis

The data were analyzed using GraphPad Prism, version 8.0, by two-way analysis of variance (ANOVA) with Tukey’s multiple-comparison test or *t*-test with non-parametric tests was used where specified in the figure legends. The bars indicate the means ± standard deviations (SD). A *p*-value of less than 0.05 was considered to be statistically significant.

## 3. Results

### 3.1. IAV induces Dispersed Trans-Golgi Network (dTGN) Formation

**Figure 1:**
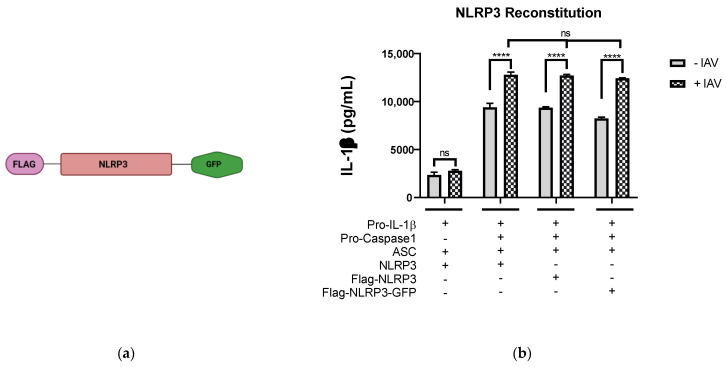

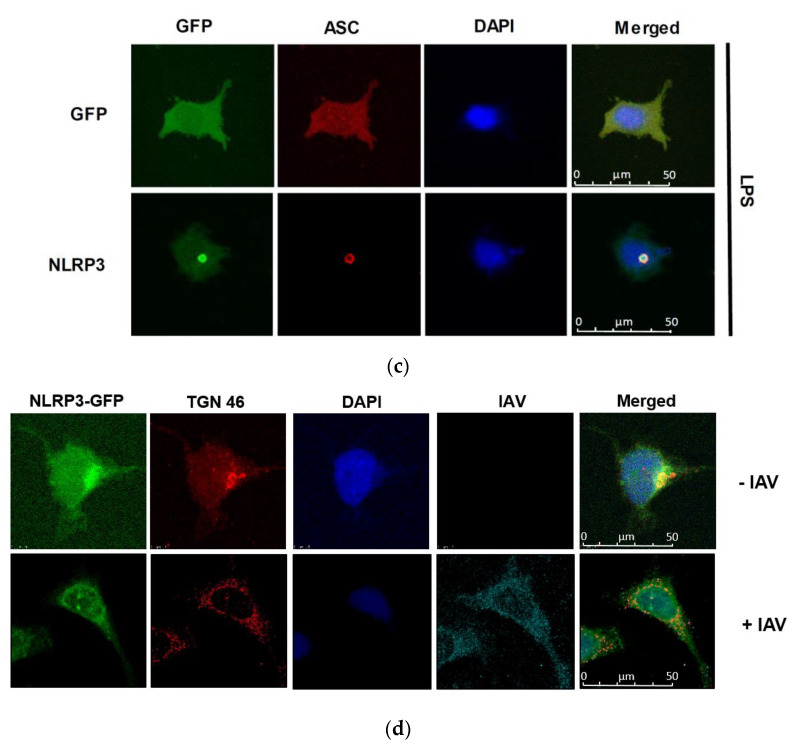
IAV infection induces the formation of dispersed trans-Golgi network. (**a**) Schematic representation of Flag-NLRP3-GFP construct. (**b**) HEK293T cells were transfected with different combinations of plasmids expressing porcine NLRP3, ASC, pro-caspase-1, and pro-IL-1β as indicated. At 12 h.p.t., the cells were mock-infected or infected with Sk02 at an MOI of 10. Porcine IL-1β from the cell-free supernatants at 12 h.p.i was measured by ELISA (two-way ANOVA; **** *p* < 0.0001; ns- Not significant). (**c**) HeLa cells were co-transfected with ASC together with either GFP or NLRP3-GFP and were stimulated with LPS (200 ng/mL) for 80 min. The cells were fixed, permeabilized, blocked, and probed with appropriate antibodies, followed by DAPI staining. GFP/NLRP3-GFP (green), ASC (red), and nucleus (blue) were visualized by confocal microscopy. Scale bar, 50 μm. (**d**) HeLa cells expressing NLRP3-GFP were infected with Sk02 at an MOI of 10. At 80 m.p.i, the cells were subjected to immunofluorescence for NLRP3-GFP (green), TGN (red), IAV (cyan), and nucleus (blue). Scale bar, 50 μm.

To find out if the IAV causes TGN dispersion, we first constructed the plasmid pFlag-NLRP3-GFP, which encodes porcine NLRP3 fused with Flag tag at the N-terminus and GFP at the C-terminus (denoted from here on as NLRP3-GFP) (Figure 1a). To test if the NLRP3-GFP construct was functionally active, we used the NLRP3 reconstitution assay established previously [16], in which HEK293T cells were co-transfected with the plasmids encoding inflammasome components (NLRP3, ASC, pro-caspase 1, and pro- IL-1β) for 24 h followed by infection with Sk02 wild type (WT) for a further 18 h. The cell-free supernatant was collected and measured for the presence of mature IL-1β by ELISA. As expected, after viral infection, the amount of mature IL-1β released from samples expressing NLRP3-GFP was similar to that in cells expressing either NLRP3 or Flag-NLRP3 (Figure 1b). Another affirmation of activated NLRP3 inflammasome is the formation of an ASC speck. To test if NLRP3-GFP could form the speck, HeLa cells were transfected with either GFP or NLRP3-GFP encoding plasmids together with ASC encoding plasmid and were stimulated by LPS, a well-known inducer of the NLRP3 inflammasome [20]. As seen in Figure 1c, the ASC speck was observed in LPS-stimulated cells expressing NLRP3-GFP, and not in cells expressing GFP. NLRP3-GFP is functionally active and can form the NLRP3 inflammasome, hence can be used for further analysis.

To test whether IAV infection would induce dTGN formation, NLRP3-GFP-expressing cells were infected with Sk02 for a period of 80 min and were observed under the confocal microscope (Figure 1d). In mock-infected cells, NLRP3-GFP shows small blobs of intact TGN near the cell nucleus, whereas the infected cells show dTGN formation described as puncta structures drifting away from the nucleus.

### 3.2. NLRP3 and ASC Are Recruited to dTGN and form Speck after IAV Infection

**Figure 2:**
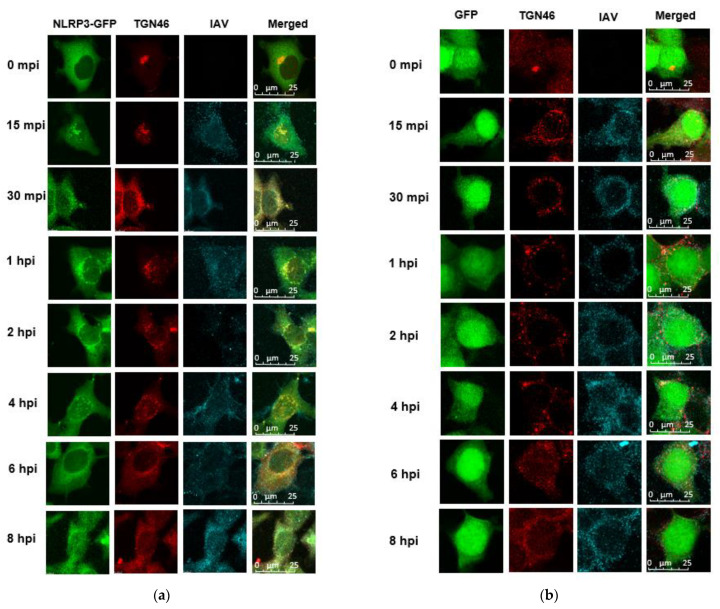

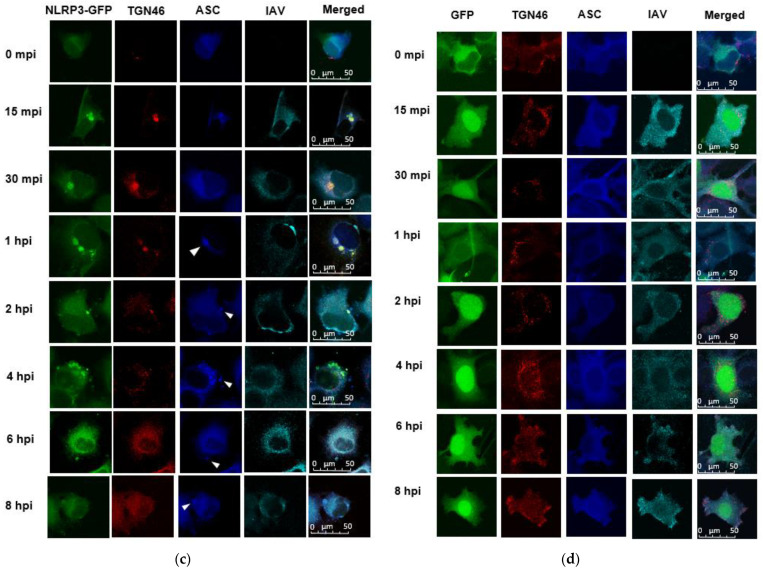
ASC is recruited to the site of dTGN-NLRP3. HeLa cells expressing (**a**) NLRP3-GFP or (**b**) GFP were infected with Sk02 at an MOI of 10. At indicated time points post-infection, cells were subjected to immunofluorescence for NLRP3 (green), TGN46 (red), and IAV (cyan). Scale bar, 25 μm. HeLa cells expressing NLRP3-GFP (**c**) or GFP (**d**) were transfected with a plasmid expressing ASC-Myc for 24 h and then were infected with Sk02 at an MOI of 10. At indicated time points, the cells were subjected to immunofluorescence microscopy for NLRP3 (green), TGN46 (red), ASC (blue), and IAV (cyan). The white arrows indicate the co-localization of NLRP3 and ASC. Scale bar, 50 μm.

To understand the kinetics of dTGN formation, NLRP3 recruitment, and ASC speck formation after IAV infection, we conducted viral infection and fluorescent microscopy. As seen in Figure 2a, at 0 min p.i., NLRP3-GFP was observed throughout the cell with the majority being in the cytoplasm in a diffused pattern—it started aggregating at 15 min p.i. and progressed until 6 h.p.i. Over these time periods, NLRP3-GFP formed puncta and were co-localized with dTGN. By 8 h.p.i., the puncta disappeared and NLRP3-GFP resumed diffusion throughout the cell. In contrast, in GFP-expressing cells, IAV infection caused TGN dispersion, however, GFP was diffused across the cell throughout the infection (Figure 2b). These data reconfirmed that IAV infection-induced TGN dispersion and suggested NLRP3 is recruited into the dTGN.

To investigate at what point the ASC is recruited to NLRP3, we transfected NLRP3-GFP cells with a plasmid expressing ASC followed by IAV infection. ASC seems to co-localize to the dTGN-NLRP3 puncta from 1 h.p.i. and continue to 8 h.p.i. (Figure 2c). In the absence of NLRP3, ASC itself was diffused across the cell and did not form any specks after IAV infection (Figure 2d). These results suggest that ASC is recruited to the site of dTGN-NLRP3.

### 3.3. M2 Protein Influences dTGN Formation and NLRP3 Inflammasome Activation

**Figure 3:**
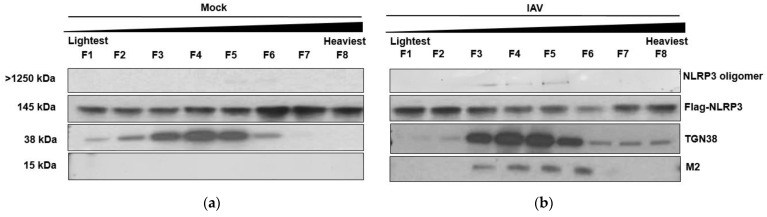

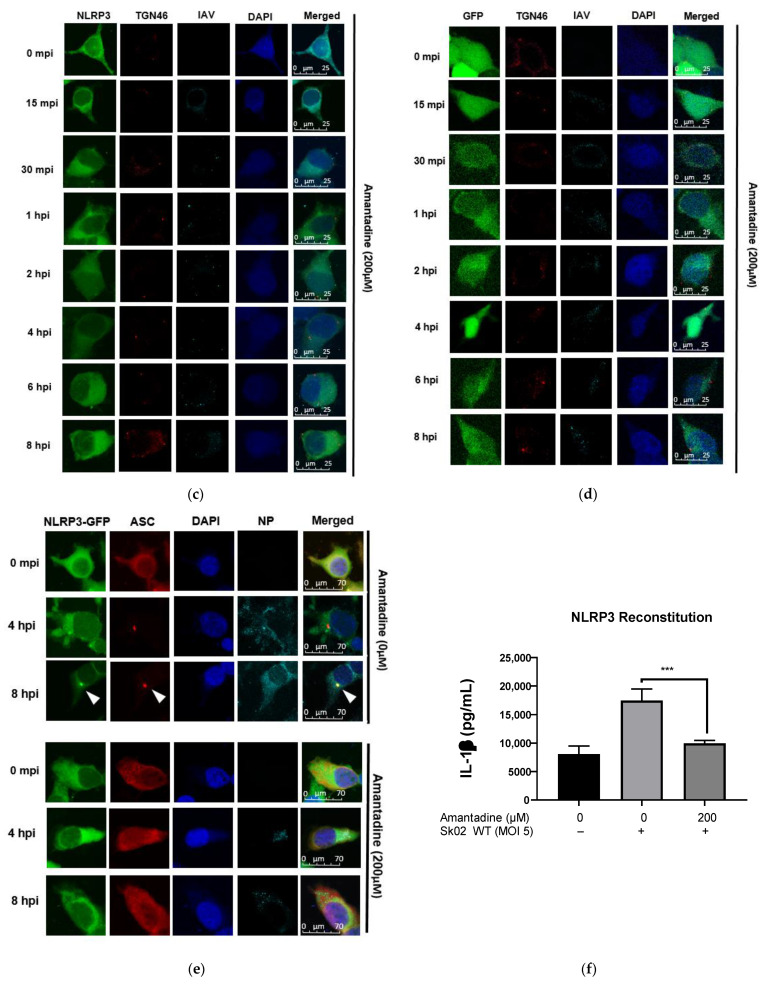
M2 Protein influences dTGN formation before NLRP3 recruitment. HEK293T cells were transfected with Flag-NLRP3 for 48 h before being mock infected (**a**) or infected with Hf09 WT at MOI of 5 for 12 h (**b**). The cells were harvested and fractionated to isolate the light membrane fraction containing TGN. The fractions were then subjected to Western blot and probed for NLRP3, TGN, and M2. The cell fraction was also subjected to a native PAGE gel to test the presence of NLRP3 oligomers (top panels). HeLa cells expressing NLRP3-GFP (**c**) or GFP (**d**) were treated with Amantadine (200 μM) for 1 hr before being infected with Hf09 (MOI of 10). The cells were subjected to immunofluorescent microscopy for NLRP3 (green), TGN (red), IAV (cyan), and nucleus (blue). Scale bar, 25 μm (**e**) Cells expressing NLRP3-GFP were transfected with ASC-Myc for 12 h. The cells were then infected with Sk02 WT at an MOI of 10 in the presence or absence of Amantadine (200 μM). The cells were fixed at indicated time points and were subjected to immunofluorescent microscopy for NLRP3 (green), ASC (red), NP (cyan), and nucleus (blue). Scale bar, 70 μm; white arrows indicate ASC speck. (**f**) HEK293T was transfected with the various components of the NLRP3 inflammasome namely, NLRP3, ASC, pro-caspase-1, and pro-1L-1β, and incubated for 12 h. The media was then changed to basal DMEM containing 200 μM Amantadine and incubated for 1 hr before being infected with Sk02 WT (MOI of 5). The cell-free supernatant was harvested at 12 h.p.i. and the level of mature IL-1β released was measured by IL-1β ELISA (*t*-test, *** *p* = 0.0004).

The M2 protein is an integrated viral membrane protein that functions as an ion channel and is responsible for acidification of the endosome upon endocytosis [21,22]. The M2 protein becomes active while moving along the exocytic pathway and equilibrates the pH between the lumen of TGN and the cytoplasm [23]. We hypothesized that the M2 protein is involved in the induction of TGN dispersion, leading to the NLRP3 recruitment and inflammasome activation. We first examined whether IAV infection would induce NLRP3 oligomerization in the TGN and whether they are associated with M2 protein. Here, Flag-NLRP3-expressing cells were infected by wild-type Hf09 for 4 h. The cells were harvested and fractionated to separate light membrane fractions containing TGN organelles by ultra-centrifugation. We observed that TGN is most abundantly detected in fractions 1–6 in mock-infected cells (Figure 3a) and fractions 3–8 in infected cells, fraction 1 being the lightest and fraction 8 being the heaviest. In concomitant, M2 and NLRP3 oligomers were co-migrated with the TGN in infected cells (Figure 3b). In contrast, NLRP3 oligomers were not observed in mock-infected cells. Next, we assessed if blocking the M2 ion channel function had any effect on the formation of dTGN. For this, we used Amantadine, which is a known inhibitor of the M2 ion channel protein. Amantadine binds to the transmembrane domain of M2, the domain, and prevents the activation of M2 [24]. According to our previous study, the NS1 protein of Hf09 is known to suppress the activation of the NLRP3 inflammasome through inhibiting ASC ubiquitination, whereas the Sk02 strain does not have such an effect [16]. Therefore, we used the Sk02 virus for this part of the experiment; GFP or NLRP3-GFP-expressing cells were infected with Sk02 WT virus at an MOI of 10 in the presence of amantadine, a known M2 ion channel inhibitor [25]. Dispersion of the TGN was not observed in cells expressing either NLRP3-GFP (Figure 3c) or GFP (Figure 3d) throughout the infection. We further tested the effect of amantadine on ASC speck formation, which is an indicator of the NLRP3 inflammasome activation. After viral infection, ASC specks were observed in untreated cells, however, were not seen in cells treated with amantadine (Figure 3e). We also quantified the level of NLRP3 activation by performing an IL-1β ELISA. Upon blocking M2 function by amantadine, there was a drastic decrease in IL-1β level in IAV-infected cells in comparison to that in the absence of amantadine (Figure 3f). All the data indicate that M2 is important for the formation of dTGN and the activation of NLRP3.

### 3.4. His37 and Trp41 in M2 Are Important for dTGN Formation and NLRP3 Conformational Change

**Figure 4:**
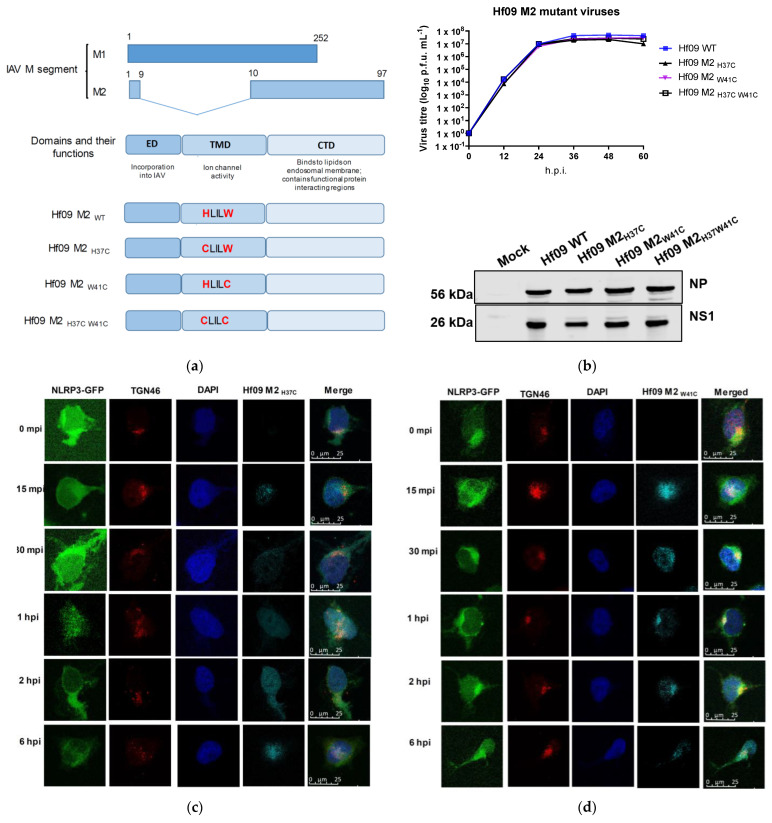

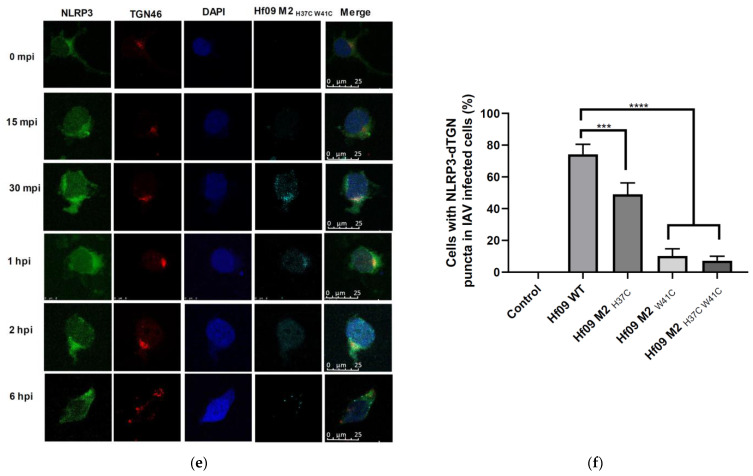
IAV M2 ion channel protein activates NLRP3. (**a**) Schematic representation of M segment-encoded proteins, as well as the mutations made on M2 protein. (**b**) MDCK-M2 cells were infected with WT or one of the M2 defective Hf09 viruses at an MOI of 0.001. The supernatant was collected every 12 h and was subjected to plaque assays to measure the viral titers. Viral protein expression was determined in the infected cells harvested at 36 h.p.i. by Western blotting. Cells expressing NLRP3-GFP were infected with Hf09 M2_H37C_ (**c**), Hf09 M2_W41C_ (**d**), or Hf09 M2_H37C-W41C_ (**e**) at an MOI of 10. At the indicated time points, the cells were fixed and subjected to immunofluorescent microscopy for NLRP3-GFP (green), TGN (red), IAV (cyan), and nucleus (blue). Scale bar, 25 μm. (**f**) The number of cells showing dTGN was counted from 100 IAV-infected cells (1 h.p.i.) in 5 different areas. (two-sided *t*-test, *** *p* = 0.0004; **** *p* < 0.0001).

To further investigate the role of M2 in TGN dispersion and NLRP3 inflammasome activation, we generated three mutant influenza viruses with functionally inactive M2 protein [11,26]. These mutations are present in the transmembrane domain of the M2 protein yet do not change the amino acids sequence of the M1 protein (Figure 4a). The transmembrane domain of the M2 protein forms a channel pore, and His37 and Trp41 are located inside the pore. While His37 is a sensor for ion selectivity, Trp41 is the gate [25,27]. The mutant viruses, namely Hf09 M2_H37C_, Hf09 M2_W41C_, and Hf09 M2_H37C-W41C_ bear a single mutation at residue His37, Trp41, or double mutations at His37 and Trp41 on the M2 protein, respectively. The viruses were first characterized for their ability to replicate in an MDCK-M2 cell line. All the mutant viruses replicated to similar titers and exhibited similar growth kinetics as did the WT virus. Consistently, the viral early (NP) and late protein (NS1) were synthesized to similar levels between the mutant viruses and the WT virus in MDCK-M2 cells (Figure 4b). These data demonstrate that the mutant viruses did not have any unwanted mutations that impaired viral replication other than the desired ones.

**Figure 5:**
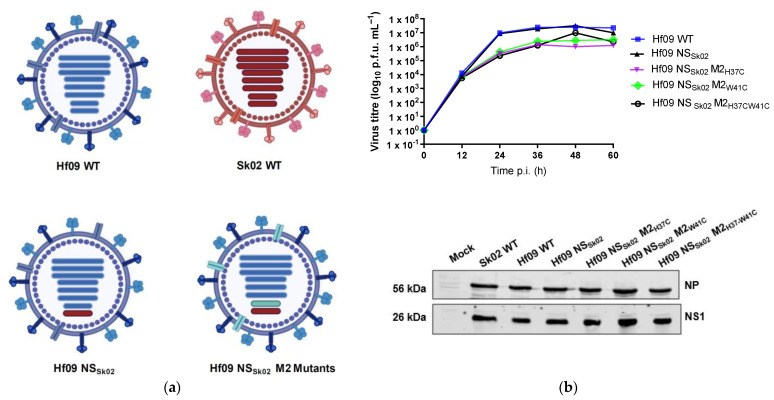
Generation of Hf09 NS_Sk02_ virus reassortants. (**a**) Schematic diagram of genome composition of WT and mutant viruses. Hf09 NS_SK02_ virus has the background of Hf09 WT (Blue) with NS segment replaced with that from Sk02 (Red) virus. Hf09 NS_Sk02_ M2 mutant viruses (turquoise) are derivatives of Hf09 NS_SK02_ wherein the 7th segment contains various mutations. (**b**) MDCK- M2 cells were infected with Hf09 NS_Sk02_ WT and the respective Hf09 NS_Sk02_ M2 mutant viruses at an MOI of 0.001. The supernatant collected at different time points was subjected to plaque assay. Viral protein expression from infected cells (MOI of 1, at 36 h.p.i.) was determined by Western blotting (lower panel) (p.f.u.—plaque forming unit; time p.i.—Time post infection).

We then tested the ability of these M2 mutant viruses to induce dispersion of TGN. While dTGN and NLRP3 granules could be found in Hf09 M2_H37C_-infected cells (Figure 4c), they were less frequently observed in Hf09 M2_W41C_- or Hf09 M2_H37C-W41—_infected cells, where TGN was found to be compact in the perinuclear region (Figure 4d,e). To quantify the number of cells displaying dTGN, we counted 100 viral-infected cells and found at 1 h.p.i, 74.2% of Hf09 WT viral-infected cells showed NLRP3-dTGN puncta, whereas 49% of Hf09 M2_H37C_-infected cells, 10.2% Hf09 M2_W41C_ and 7.2% Hf09 M2_H37C-W41C_-infected cells showed dTGN (Figure 4f). Our previous study showed that the NS1 protein of Hf09 WT virus suppresses NLRP3 activation and IL-1β production [16], to eliminate this effect, we generated a new series of reassortants in the background of the Hf09 M2 mutants where the NS segment was replaced with that of Sk02 virus, whose NS1 protein does not inhibit NLRP3 activity (Figure 5a). The Hf09-NS_Sk02_ virus comprises seven segments from the Hf09 strain and the NS segment from Sk02, in the MDCK-M2 cell line it replicated as well as the Hf09 WT virus. All the other reassortants containing a single mutation or double mutations in the M2 protein replicated slightly slower than Hf09-NS_Sk02__,_ however, the levels of NP protein and NS1 protein produced by the reassortant mutants were similar to that of Hf09-NS_Sk02_ (Figure 5b).

**Figure 6:**
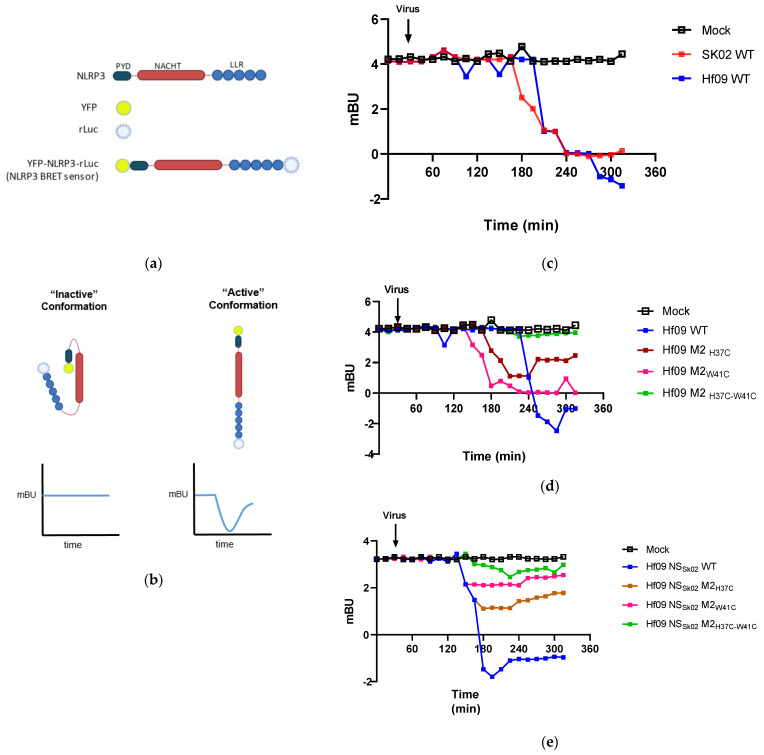
IAV induces NLRP3 conformational change upon infection. (**a**) Schematic representation illustrating the various components of the NLRP3 BRET sensor and (**b**) how the change in conformation of NLRP3 affects BRET signals (the two blue lines indicate BRET signals). HEK293T cells were transfected with pcDNA3.1- YFP-NLRP3-Luc for 24 h. The cells were then treated with Coelanterazine-h (5 μM). After 30 min, cells were infected with the following series of viruses at MOI of 1: Hf09 and Sk02 wild-type viruses (**c**) Hf09 virus and its derivatives containing M2 mutation (**d**), or Hf09 NS_Sk02_ virus and its derivatives containing M2 mutation (**e**). The signal was recorded every 15 min and BRET signal is expressed as mBU (milli-BRET unit). The black arrow represents when the virus was added.

Using these viruses, we conducted a *bioluminescence resonance energy transfer* (BRET) assay to analyze how the M2 mutations would affect the NLRP3 conformational change. It is known that upon sensing the presence of DAMP or PAMP signals, NLRP3 changes its confirmation from the closed inactive state to the open active state [28]. For the BRET assay, HEK293T cells were transfected with a plasmid encoding NLRP3 BRET sensor (Figure 6a). In the closed or inactive conformation, the distance between luciferase and the YFP is less than 10 nm, this allows the donor (luciferase) to transfer non-radiative energy to the acceptor (YFP) resulting in fluorescence emission at a characteristic wavelength of 530 nm. The energy emitted by the acceptor relative to that emitted by the donor is termed the BRET signal which is expressed as milli-BRET Unit (mBU) [29]. A decrease in the BRET signal indicates a change in the conformation of the NLRP3 leading to the open or active conformation (Figure 6b). We first examined the BRET signal in response to wild-type Sk02 and Hf09 infection (Figure 6c). We observed a 4 to 5 mBU dip over the period of infection, confirming IAV infection caused a conformational change in NLRP3. We further tested the degree of conformational change in NLRP3 in response to infection by IAV M2 mutants. We observed that Hf09 M2_H37C_, Hf09 M2_W41C_, and Hf09 M2_H37C-W41C_ all caused a reduced degree of mBU dip as compared to the WT Hf09 virus infection, of which, Hf09 M2_H37C-W41C_ led to a least mBU dip of 1 (Figure 6d). Similarly, analysis of the reassortant Hf09 viruses carrying the NS gene derived from Sk02 showed a similar pattern (Figure 6e), compared to the Hf09 NS_Sk02_ WT whose infection led to a dip of over 4 mBU, mutations on M2 resulted in a reduced degree of mBU dips, indicating that loss of M2 ion channel activity impairs the activation of NLRP3.

### 3.5. His37 and Trp41 in M2 Affects the IL-1β Production

**Figure 7:**
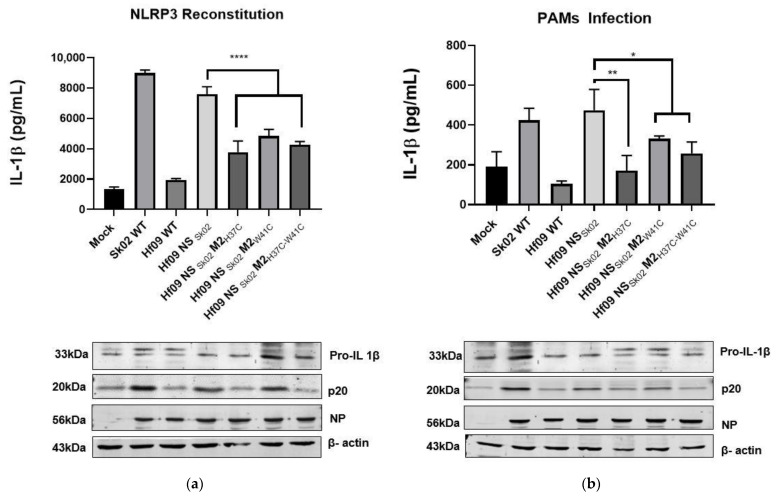
His37 and Trp41 in M2 affect IL-1β production. (**a**) HEK293T cells were transfected with different combinations of plasmids expressing porcine NLRP3, ASC, pro-caspase-1, and pro-IL-1β. At 12 h.p.t., the cells were mock-infected or infected with the viruses as indicated at an MOI of 5. Porcine IL-1β from the cell-free supernatants at 12 h.p.i. was measured by ELISA. The expression of pro-IL-1β, the active caspase-1, p20, NP, and β-actin was measured by Western blotting in the cell lysates (*t*-test, **** *p* < 0.0001). (**b**) PAMs were infected with various viruses as indicated at an MOI of 1 for 24 h. The cell-free supernatant was collected to measure the IL-1β level by ELISA and the cell pellet was lysed and checked for the level of pro-IL-1β, p20, NP, and β-actin by Western blotting (*t*-test, ** *p* = 0.0033; * *p* = 0.0339).

To test how the mutations on M2 would impact the ability of the virus to induce IL-1β because of NLRP3 activation, we conducted an NLRP3 reconstitution assay followed by infection (Figure 7a) as well as PAMs infection with the respective virus (Figure 7b) and measured the production of IL-1β. In agreement with our previous results, in both reconstitution and PAMs infection assays, Sk02 WT infection induced a significantly high level of IL-1β production, however, Hf09 WT infection produced only a similar level of IL-1β as in the mock-infected cells. As expected, replacing the Hf09 NS segment with that of Sk02 (Hf09 NS_Sk02_) could restore the ability to induce IL-1β production. Mutations on the M2 protein, namely H37C, W41C, and H37C/W41C could significantly reduce IL-1β production. Western blotting results showed the reduction in mature IL-1β levels was not due to the decreased pro-IL-1β synthesis nor the insufficient viral replication. The level of p20, as a result of NLRP3 inflammasome activation and the cleavage of caspase-1, is consistent with the IL-1β level detected by ELISA, indicating that changes in IL-1β were attributable to NLRP3 inflammasome activity.

## 4. Discussion

Extensive studies have investigated the cellular organelles’ contribution to the activation of NLRP3 inflammasome. Disruption of lysosomes resulted in the activation of the NLRP3 pathway through the release of active cathepsin B [30]. Loss of mitochondria membrane potential and the association of NLRP3 with mitochondrial antiviral signaling protein (MAVS) promote inflammasome activation and the production of IL-1β [31,32]. Recently, TGN has been identified as another platform where NLRP3 and its downstream adaptor proteins are recruited and assembled into active inflammasome. Various NLRP3 stimuli such as nigericin and ATP promoted the disassembly of the TGN into puncta-like structures. The NLRP3 was observed to be recruited to the phospholipid phosphatidylinositol-4-phosphate on dTGN, where it aggregated [12]. NLRP3 aggregation is known to be essential for downstream ASC oligomerization and caspase-1 activation [12]. Golgi fragmentation was previously reported upon various virus infections. Poliovirus induced disruption of the Golgi complex [33]; African swine fever virus caused loss of the Golgi structure in infected cells [34]. Viral infection-induced dispersion of TGN was proposed to be associated with viral pathogenesis; however, viral-induced dTGN has never been examined in terms of its role in inflammasome activation.

IAV infection-induced IL-1β and IL-18 production through caspase-1 dependent manner has been known for decades [35], however, the exact mechanism by which IAV activates NLRP3 inflammasome is not fully understood. Several IAV proteins have been identified to be involved in activating and regulating inflammasome activity directly or indirectly. PB1-F2 protein activates NLRP3 through various mechanisms: the aggregated form of the C-terminal region of the PB1-F2 protein has been shown to activate NLRP3 through mitophagy [10]; the translocation of the PB1-F2 protein to the mitochondria promotes the production of mtROS and the release of mitochondrial DNA (mtDNA), which activates NLRP3 [36]. It is interesting to note that endocytosis of extracellular PB1-F2 from H7N9 IAV causes hyperactivation of the NLRP3 inflammasome, whereas internally expressed PB1-F2 has the opposite effect. It has been shown that this selective suppression occurs through the MAVS-dependent NLRP3 inflammasome activation pathway [37]. The IAV M2 protein was also shown to activate the inflammasome by regulating ion flux [11] or stimulating cytosolic mitochondrial DNA release [38].

Prompted by these studies, we are interested in investigating whether IAV infection would induce TGN dispersion and whether dTGN contributes to one of the mechanisms by which IAV activates the NLRP3 inflammasome. We observed TGN dispersed into puncta-like structures over the course of IAV infection (15 m.p.i up to 6 h.p.i.). dTGN was observed not only in NLRP3-GFP-expressing cells but also in GFP-expressing cells suggesting NLRP3 is recruited as a result of TGN dispersion. NLRP3-ASC speck formation and the co-migration of oligomerized NLRP3 with TGN demonstrated that NLRP3 is not only recruited to the TGN but also activated. These results are in line with findings reported by Chen and Chen using nigericin as a stimulus [12]. In terms of how NLRP3 is recruited to the dTGN, it was found that binding of the polybasic region (KKKK) in NLRP3 to the negatively charged phospholipid PtdIns4P, which is enriched in dTGN, is essential for NLRP3 recruitment and activation [12]. Whether IAV infection employs a similar mechanism by which NLRP3 is recruited to the dTGN warrants further investigation.

The M2 protein is a well-known ion channel protein. Upon IAV endocytosis, when the M2 protein finds an endosome under acidic conditions of pH 6, it is activated and leads to the fusion of the endosomal membrane with the viral membrane resulting in the release of the viral genetic material into the cytoplasm [21]. Upon the translation and transcription of the viral genetic material, newly formed M2 protein moves through the Golgi network for its post-translational modifications and upon reaching the TGN, seem to be activated, as the pH in the TGN is also 6, which is the favorable condition for the activation of M2. In turn, as M2 has a proton selective channel function, its expression affects the pH of the acidified cellular compartments including TGN [39,40]. We, therefore, hypothesized that the M2 protein is responsible for TGN dispersion.

In the fractionation assay, we observed that the fractions containing the most abundant TGN also contained M2 and NLRP3 oligomers. This indicated the presence of the M2 ion channel protein in the dTGN-NLRP3 fractions. Blocking the function of the M2 ion channel activity by amantadine leads to the reduction in dTGN, ASC specks, and mature IL-1β. These data provide primary evidence that M2 plays an essential role in inducing dTGN and inflammasome activation. The amino acid histidine at position 37 and tryptophan at position 41 are the main factors dictating the ion channel function of M2 [41]. To seek direct evidence that M2 regulates TGN dispersion through its ion channel activity, we mutated these amino acids into cysteine residues [26] to make the IAV mutants deficient in M2 ion channel function. The initial mutant viruses were constructed in the background of the Hf09 virus (Figure 4). We observed, that while mutant viruses significantly reduced the formation of dTGN-NLRP3, mutation at His37 seems to have a less profound effect than Trp41. This might be explained that His37 is important for the selectivity of the ions for subsequent transport of the ions; the main gate for the ions to travel through the protein was provided by the Trp41 [41].

The NS1 protein of the Hf09 virus is known to suppress the release of mature IL-1β, whereas the NS1 protein of the Sk02 WT virus does not possess this feature [16]. Mechanistically, the NS1 C-terminus of the 2009 pandemic stain Hf09 inhibits ASC speck formation by suppressing the ubiquitination of ASC [16]. We, therefore, constructed a second series of mutant viruses wherein the 8th segment of Hf09 is replaced by that of Sk02 (Figure 5). This would help us to observe the effects of the M2 mutation without much hindrance from the effects of the NS1 protein inhibitory function. We observed that all mutant viruses induced decreased levels of IL-1β in comparison to their corresponding WT virus. The reduction in IL-1β is a direct result of less NLRP3 activity as measured by the BRET assay.

**Figure 8:**
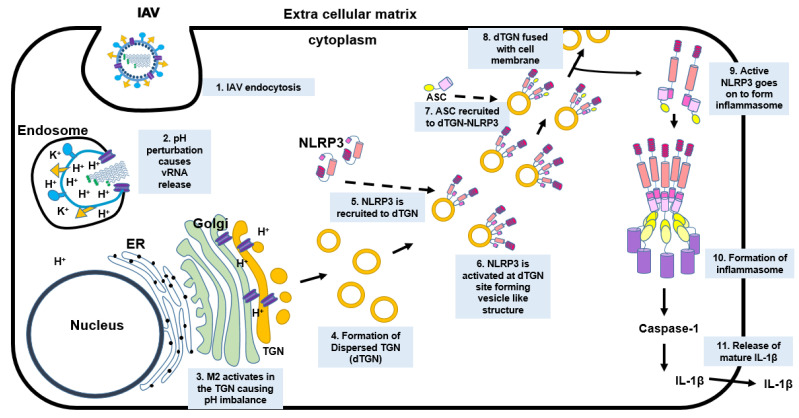
Schematic representation of the working model. Upon endocytosis of IAV, the pH of the endosome (pH 6) activates the IAV M2 ion channel protein leading to the release of vRNP into the cytoplasm. The vRNP then translocates into the nucleus to undergo transcription and translation. The newly translated M2 protein travels from the ER through the Golgi apparatus and reaches TGN. The pH of the TGN (pH 6) causes a pH disparity in the pH-sensitive organelle which leads to the dispersion of the TGN (dTGN). The NLRP3 molecules then travel to the newly formed dTGN where the NLRP3 undergoes conformational changes, oligomerization, and becomes active. The ASC molecule is then recruited to the site of dTGN-NLRP3, where the NLRP3 binds to ASC from its PYD domain. The NLRP3–ASC complex then leaves the dTGN and goes on to form the inflammasome, while the dTGN goes on to fuse with the cell membrane. The active NLRP3 inflammasome then leads to the production of active caspase-1 which then converts pro-IL-1β to mature IL-1β. The mature IL-1β is then released into the extracellular matrix, which then signals other immune cells to travel to the site of infection.

In conclusion, we have shown that IAV M2 protein regulates TGN dispersion through its ion channel function. NLRP3 is recruited to the dTGN, where NLRP3 is oligomerized, undergoes a conformational change, and recruits ASC. As a result, the inflammasome is activated leading to the production of IL-1β (Figure 8). While the production of IL-1β is beneficial for the host immune defense, dysregulation of the innate immune response may contribute to viral pathogenesis. Our finding contributes to a novel mechanism by which IAV infection activates the NLRP3 inflammasome.

## Data Availability

Not applicable.

## References

[1] Mogensen T.H. (2009). Pathogen Recognition and Inflammatory Signaling in Innate Immune Defenses. Clin. Microbiol. Rev..

[2] Amarante-Mendes G.P., Adjemian S., Branco L.M., Zanetti L., Weinlich R., Bortoluci K.R. (2018). Pattern Recognition Receptors and the Host Cell Death Molecular Machinery. Front. Immunol..

[3] Swanson K.V., Deng M., Ting J.P.-Y. (2019). The NLRP3 inflammasome: Molecular activation and regulation to therapeutics. Nat. Rev. Immunol..

[4] Malik A., Kanneganti T.-D. (2017). Inflammasome activation and assembly at a glance. J. Cell Sci..

[5] Kelley N., Jeltema D., Duan Y., He Y. (2019). The NLRP3 Inflammasome: An Overview of Mechanisms of Activation and Regulation. Int. J. Mol. Sci..

[6] Stutz A., Horvath G.L., Monks B.G., Latz E. (2013). ASC Speck Formation as a Readout for Inflammasome Activation. Methods Mol. Biol..

[7] Schroder K., Zhou R., Tschopp J. (2010). The NLRP3 inflammasome: A sensor for metabolic danger?. Science.

[8] Iwasaki A., Pillai P.S. (2014). Innate immunity to influenza virus infection. Nat. Rev. Immunol..

[9] Sarvestani S.T., McAuley J.L. (2017). The role of the NLRP3 inflammasome in regulation of antiviral responses to influenza A virus infection. Antivir. Res..

[10] McAuley J.L., Tate M., MacKenzie-Kludas C.J., Pinar A., Zeng W., Stutz A., Latz E., Brown L., Mansell A. (2013). Activation of the NLRP3 Inflammasome by IAV Virulence Protein PB1-F2 Contributes to Severe Pathophysiology and Disease. PLoS Pathog..

[11] Ichinohe T., Pang I.K.-S., Iwasaki A. (2010). Influenza virus activates inflammasomes via its intracellular M_2_ ion channel. Nat. Immunol..

[12] Chen J., Chen Z.J. (2018). PtdIns4P on dispersed trans-Golgi network mediates NLRP3 inflammasome activation. Nature.

[13] Weingartl H., Sabara M., Pasick J., van Moorlehem E., Babiuk L. (2002). Continuous porcine cell lines developed from alveolar macrophages: Partial characterization and virus susceptibility. J. Virol. Methods.

[14] Liu G., Lu Y., Raman S.N.T., Xu F., Wu Q., Li Z., Brownlie R., Liu Q., Zhou Y. (2018). Nuclear-resident RIG-I senses viral replication inducing antiviral immunity. Nat. Commun..

[15] Gaba A., Xu F., Lu Y., Park H.-S., Liu G., Zhou Y. (2019). The NS1 Protein of Influenza A Virus Participates in Necroptosis by Interacting with MLKL and Increasing Its Oligomerization and Membrane Translocation. J. Virol..

[16] Park H.-S., Liu G., Raman S.N.T., Landreth S.L., Liu Q., Zhou Y. (2018). NS1 Protein of 2009 Pandemic Influenza A Virus Inhibits Porcine NLRP3 Inflammasome-Mediated Interleukin-1 Beta Production by Suppressing ASC Ubiquitination. J. Virol..

[17] Hoffmann E., Neumann G., Kawaoka Y., Hobom G., Webster R.G. (2000). A DNA transfection system for generation of influenza A virus from eight plasmids. Proc. Natl. Acad. Sci. USA.

[18] Shin Y.-K., Liu Q., Tikoo S.K., Babiuk L.A., Zhou Y. (2007). Effect of the phosphatidylinositol 3-kinase/Akt pathway on influenza A virus propagation. J. Gen. Virol..

[19] Duan Y., Zhang L., Angosto-Bazarra D., Pelegrín P., Núñez G., He Y. (2020). RACK1 Mediates NLRP3 Inflammasome Activation by Promoting NLRP3 Active Conformation and Inflammasome Assembly. Cell Rep..

[20] Gritsenko A., Yu S., Martin-Sanchez F., Diaz-Del-Olmo I., Nichols E.-M., Davis D., Brough D., Lopez-Castejon G. (2020). Priming Is Dispensable for NLRP3 Inflammasome Activation in Human Monocytes In Vitro. Front. Immunol..

[21] Manzoor R., Igarashi M., Takada A. (2017). Influenza A Virus M_2_ Protein: Roles from Ingress to Egress. Int. J. Mol. Sci..

[22] Dou D., Revol R., Östbye H., Wang H., Daniels R. (2018). Influenza A Virus Cell Entry, Replication, Virion Assembly and Movement. Front. Immunol..

[23] Shimbo K., Brassard D., Lamb R., Pinto L. (1996). Ion selectivity and activation of the M_2_ ion channel of influenza virus. Biophys. J..

[24] Cady S., Schmidt-Rohr K., Wang J., Soto C.S., DeGrado W.F., Hong M. (2010). Structure of the amantadine binding site of influenza M2 proton channels in lipid bilayers. Nature.

[25] Pinto L.H., Holsinger L.J., Lamb R.A. (1992). Influenza virus M_2_ protein has ion channel activity. Cell.

[26] Shuck K., Lamb R.A., Pinto L.H. (2000). Analysis of the Pore Structure of the Influenza A Virus M_2_ Ion Channel by the Substituted-Cysteine Accessibility Method. J. Virol..

[27] Tang Y., Zaitseva F., Lamb R.A., Pinto L.H. (2002). The Gate of the Influenza Virus M2 Proton Channel Is Formed by a Single Tryptophan Residue. J. Biol. Chem..

[28] Tapia-Abellán A., Angosto D., Banaclocha H.M., de Torre C., Cerón-Carrasco J.P., Pérez-Sánchez H., Arostegui J.I., Pelegrin P. (2019). MCC950 closes the active conformation of NLRP3 to an inactive state. Nat. Chem. Biol..

[29] Dale N.C., Johnstone E.K.M., White C.W., Pfleger K.D.G. (2019). NanoBRET: The Bright Future of Proximity-Based Assays. Front. Bioeng. Biotechnol..

[30] Hornung V., Bauernfeind F., Halle A., Samstad E.O., Kono H., Rock K.L., Fitzgerald K., Latz E. (2008). Silica crystals and aluminum salts activate the NALP3 inflammasome through phagosomal destabilization. Nat. Immunol..

[31] Park S., Juliana C., Hong S., Datta P., Hwang I., Fernandes-Alnemri T., Yu J.-W., Alnemri E.S. (2013). The Mitochondrial Antiviral Protein MAVS Associates with NLRP3 and Regulates Its Inflammasome Activity. J. Immunol..

[32] Ichinohe T., Yamazaki T., Koshiba T., Yanagi Y. (2013). Mitochondrial protein mitofusin 2 is required for NLRP3 inflammasome activation after RNA virus infection. Proc. Natl. Acad. Sci. USA.

[33] Beske O., Reichelt M., Taylor M., Kirkegaard K., Andino R. (2007). Poliovirus infection blocks ERGIC-to-Golgi trafficking and induces microtubule-dependent disruption of the Golgi complex. J. Cell Sci..

[34] McCrossan M., Windsor M., Ponnambalam S., Armstrong J., Wileman T. (2001). The trans Golgi Network Is Lost from Cells Infected with African Swine Fever Virus. J. Virol..

[35] Pirhonen J., Sareneva T., Kurimoto M., Julkunen I., Matikainen S. (1999). Virus infection activates IL-1 beta and IL-18 production in human macrophages by a caspase-1-dependent pathway. J. Immunol..

[36] Wang R., Zhu Y., Ren C., Yang S., Tian S., Chen H., Jin M., Zhou H. (2020). Influenza A virus protein PB1-F2 impairs innate immunity by inducing mitophagy. Autophagy.

[37] Cheung P.H., Ye Z., Lee T.T., Chen H., Chan C.P., Jin D. (2020). PB1-F2 protein of highly pathogenic influenza A (H7N9) virus selectively suppresses RNA-induced NLRP3 inflammasome activation through inhibition of MAVS-NLRP3 interaction. J. Leukoc. Biol..

[38] Moriyama M., Koshiba T., Ichinohe T. (2019). Influenza A virus M2 protein triggers mitochondrial DNA-mediated antiviral immune responses. Nat. Commun..

[39] Sakaguchi T., Leser G.P., Lamb R.A. (1996). The ion channel activity of the influenza virus M2 protein affects transport through the Golgi apparatus. J. Cell Biol..

[40] Henkel J.R., Popovich J.L., Gibson G.A., Watkins S.C., Weisz O.A. (1999). Selective perturbation of early endosome and/or trans-Golgi network pH but not lysosome pH by dose-dependent expression of influenza M2 protein. J. Biol. Chem..

[41] Pinto L.H., Lamb R.A. (2006). The M2 Proton Channels of Influenza A and B Viruses. J. Biol. Chem..

